# Cajal, the neuronal theory and the idea of brain plasticity

**DOI:** 10.3389/fnana.2024.1331666

**Published:** 2024-02-19

**Authors:** Jairo A. Rozo, Irene Martínez-Gallego, Antonio Rodríguez-Moreno

**Affiliations:** ^1^Laboratory of Cellular Neuroscience and Plasticity, Universidad Pablo de Olavide, Seville, Spain; ^2^Iván Pávlov Laboratory, Faculty of Psychology, Los Libertadores University Foundation, Bogotá, Colombia

**Keywords:** Santiago Ramón y Cajal, neuronal theory, plasticity, history, legacy

## Abstract

This paper reviews the importance of Cajal’s neuronal theory (the Neuron Doctrine) and the origin and importance of the idea of brain plasticity that emerges from this theory. We first comment on the main Cajal’s discoveries that gave rise and confirmed his Neuron Doctrine: the improvement of staining techniques, his approach to morphological laws, the concepts of dynamic polarisation, neurogenesis and neurotrophic theory, his first discoveries of the nerve cell as an independent cell, his research on degeneration and regeneration and his fight against reticularism. Second, we review Cajal’s ideas on brain plasticity and the years in which they were published, to finally focus on the debate on the origin of the term plasticity and its conceptual meaning, and the originality of Cajal’s proposal compared to those of other authors of the time.

## Introduction

Santiago Ramón y Cajal (1852–1934) discoveries have been incorporated into the scientific knowledge of our time. In previous articles we have highlighted the importance of some aspects of his work (Rozo and Rodríguez-Moreno, 2014, 2015a,b; Rozo et al., 2017; Mateos-Aparicio and Rodríguez-Moreno, 2019; Rozo et al., 2022). Here we briefly introduce Cajal’s general influence in Neuroscience and highlight Cajal’s neuronal theory and the origin of the idea of brain and neuronal plasticity.

The work of Cajal, developed more than a century ago, founded modern neuroscience and thus, is still relevant and cited in the present days (DeFelipe, 2002; Alonso and De Carlos, 2018). For instance, according to a study by Gamundí et al. (2006), which analysed the impact of Cajal’s scientific work between 1945 and 2004, Cajal received a total of 17.259 citations during this period, his most cited works being: *Textura del Sistema Nervioso de los Hombres y los Vertebrados (Texture of the Nervous System of Humans and Vertebrates)* with 7.651 citations and *Degeneración y Regeneración del Sistema Nervioso (Degeneration and Regeneration of the Nervous System)* with 2.509 citations. Comparatively, Cajal’s total number of citations was three times those of Sherrington (5.743) and 17 times those of Golgi (965) in the same period.

The importance of the work of Ramón y Cajal has already been highlighted by different relevant neuroscientists. For instance, Pío del Río Hortega, the discoverer of the Microglia (Del Río Hortega, 1939) and an active member of the long-lived “Escuela Histológica Española” (Spanish Histological School) (Del Río Hortega and Estable, 1944), that worked with Cajal from 1917 to 1920 said:

In order to know the extension and depth of Cajal’s work in its multiple physiognomy it is necessary to look at past times and then, comparatively, at the present time. That is to say, to look for the contrast between intentions and achievements. It is necessary to travel with Cajal’s thoughts in his youth in order to pinpoint the formation of his powerful will, and to follow him in his younger years to see his vocation awaken, and to accompany him in his mature years to witness the dawn of his patriotism^1^ (p.15).

Or we can return to Sotelo’s words to understand the influence of Cajal as the father of the neuronal theory:

Cajal succeeds in unifying the tissues of the organism, and proves that the brain is formed like the rest of the body by independent units called cells. His studies on the architectural organisation of the brain and his prophetic predictions about its function, and his laws of dynamic polarisation have formed the basis of neuroanatomy, neurophysiology, neuropathology and what Cajal called “rational psychology.” All the branches, from molecular to behavioural, that form the neurosciences are still based on this foundation (Sotelo, 2008, p. 201).

We can also remember the words that his disciple Jorge Francisco Tello wrote in the prologue of the eleventh edition of *Elementos de Histología Normal y de Técnica Micrográfica (Elements of Normal Histology and Micrographic Technique):*

Cajal’s contribution to Histology is of enormous importance; it can be said that there is no chapter of this science in whose clarification he has not intervened to a great extent. But in the histology of the nervous system his contribution was decisive, having revolutionized current ideas about its constitution in 1888 and collected, in fifty years of tireless and brilliant work, an enormous mass of discoveries, many of them gathered in two large volumes of his Histología del Sistema Nervioso del Hombre y de los Vertebrados (Histology of the Nervous System of Man and Vertebrates); not a few scattered in three hundred original monographs published in national and foreign magazines, especially in five volumes of the Revista Trimestral Micrográfica (Micrographic Quarterly Journal) and in the first thirty works of Trabajos de Laboratorio de Investigaciones Biomédicas (Biological Researches Laboratory works) (Durfort, 2006, p. 144).

One of the many ideas that emerge from Cajal’s Neuron Doctrine in which Cajal clearly influenced present research is the plasticity of nerve cells, through what he called at the time “cerebral gymnastics” (Cajal, 1892, 1894a,b). Cajal believed that the cortical architecture was not a fixed structure but a dynamic and variable one related to mental processes (revised in DeFelipe, 2002, 2006a,b, 2007; Azmitia, 2007; Berlucchi and Buchtel, 2009).

## Cajal and the neuronal theory

In 1887, thanks to the influence of his friend Luis Simarro, Cajal learned about the works and writings of Camilo Golgi and his chrome-silver staining, which at that time was unstable and sometimes offered contradictory results. This was a new technique for the study of the nervous system that made possible to visualise neurons by staining them black (*reazione nera* or *black reaction*). Simarro was fundamental for Cajal’s works in two important moments. Firstly, by introducing him to Golgi’s works and his staining method in 1887. Simarro showed to Cajal some very good preparations with the chromo-silver method, where nerve cells and their prolongations were stained in a precise and selective way (Cajal, 1923/1981; López-Piñero, 2000; De Carlos and Borrell, 2007). And secondly, by creating the technique of impregnation by photographic salts of silver nitrate in 1890 (Simarro, 1900), which Cajal later modified in 1903, and which led to the reduced silver nitrate method, allowing the study of the cytoplasm of the neuron (Cajal, 1910). Luis Simarro, despite being only three years older than Cajal, was a young figure of histology in Spain who had been in Paris between 1880–1885, together with Mathias Duval, Louis Antoine Ranvier, Jean Martin Charcot and Valentin Magnan (López-Piñero, 1986).

Cajal, impressed by the technique, dedicated himself to purifying, improving and stabilizing it, so that it could be easily reproduced by other scientists. For this reason, it becomes his first technical “weapon,” especially when introducing the modification that he called “*double impregnation procedure*,” which greatly improved the quality of the images of the neurohistological preparations, achieving very clear and almost constant staining (López-Piñero, 1986; Rocha, 2007). Cajal worked with this method for 15 years in the histological study of the olfactory bulb and the retina, the spinal cord, the cerebellum, the brainstem and the cerebral cortex (DeFelipe, 1999; Campos, 2006), as he described in the book he published in 1917 about the memories of his life:

My successes at that time were undoubtedly due to some improvements to the chrome-silver method, particularly the modification known as the double impregnation procedure (Cajal, 1917)

Cajal systematically studied the nervous system, not only thanks to the staining technique, but also to his way of dealing with the problem of the complexity of the nervous system. It occurred to him to approach it from the point of view of comparative anatomy and ontogenetic development, in such a way that if he analysed embryo slices (of birds and mammals) he could see the evolution of the nervous system without the complexity inherent adults. Cajal applied this rule systematically to understand the complex brain structure of higher animals. Such embryological investigations were carried out by Cajal in strict accordance with the assumptions of Darwinian morphology and, specifically, with the law of biogenetics of Fritz Müller and Ernst Haeckel, which stated that ontogeny or individual embryonic development is a recapitulation of the phylogeny or evolutionary development of the species (López-Piñero, 1986). Thank to this, he was able to formulate the individuality of nerve cells, as essential units of the nervous system, as indicated by López-Muñoz et al. (2006):

Cajal’s work also represents the definitive culmination of cell theory. The four cardinal milestones of cell theory are therefore marked by the names of Schleiden, Schwann, Virchow and Cajal. In the last polemical work of his life, Cajal notes with extraordinary lucidity that, while defending neuronism, he also defends Virchow’s old and brilliant cellular conception (p. 88).

From 1888 to 1900 Cajal published in the *Revista Trimestral de Histología Normal y Patológica*, the articles that supported the neuronal theory. Here, Cajal formulated the total autonomy and independence of nerve cells for the first time: *“each element is an absolutely autonomous physiological canton”* (Cajal, 1888a, p. 9) and observed the existence of dendritic spines: *“the surface appears bristling with short points or spines, represented by slight asperities”* (Cajal, 1888a, p. 4). Cajal also discovered that the extensions of the nerve cells terminate freely and communicate with each other by contact, not by continuity (Cajal, 1889), term that was coined as “synapse” in 1897 (Sherrington, 1897).

Cajal discovered and named dendritic spines when he was studying the cerebellum of birds using Golgi’s method in 1888 (Cajal, 1888a,b). In 1890 he also described them in the cerebral cortex and began to describe them as a typical structure that starts thin and ends in the form of a bulb. Although very important scientists of the time confirmed the finding, scientists such as Kölliker or Golgi believed that they were an artefact of the chromo-silver technique, as they had only been seen with this staining method. Cajal then worked with other staining techniques to demonstrate their existence and found in 1896 that, when using methylene blue, they could also be observed, thus eliminating the doubt as to whether they were artefacts or anatomically real (reviewed by Cid, 1985 and DeFelipe, 2006a,b, 2007). However, on this point, as on the autonomy of neurons and other hypotheses, Cajal was ahead of his time, and we had to wait 50 years for the development of electron microscopy to be able to close this chapter in our knowledge of the nervous system. We now know that the number of dendritic spines largely reflects the number of excitatory afferents that a neuron receives.

But his results and publications were totally foreign to the scholars in Europe, who viewed them with total scepticism. He had to present his preparations directly at the Congress of the German Anatomical Society in Berlin in 1889 and to take the important German scholar Kölliker almost by the hand so that he would listen to the new discoveries: nerve cells were independent, autonomous and did not form part of a diffuse network, as the reticularist theory claimed. The support of the great figure of Kölliker helped Cajal’s ideas and studies to become internationally known and to be accepted by different experts between 1890 and 1891. Among them, the Germans Wilhelm His and Heinrich Waldeyer, the Swedish Gustav Retzius, the Hungarian Mihály Lenhossék, the Belgian Arthur van Gehuchten and the French Mathías Duval (López-Piñero, 1986).

In summary, from his studies, Cajal was able to extrapolate, between 1888 and 1889, certain “morphological laws” and “laws of nerve cell connections”:

1. The collateral and terminal branches of each axis-cylinder lead to the grey matter, not through a diffuse network, as advocated by Gerlach and Golgi and most neurologists, but through free arborisations, arranged in various ways (pericellular baskets or nests, climbing branches, etc.).
2. These ramifications are intimately applied to the body and dendrites of nerve cells, establishing a contact or articulation between the receptor protoplasm and the last axonal ramuscles.
3. Since the body and dendrites of neurons are closely applied to the last roots of the axon-cylinders, it is necessary to admit that the soma and the protoplasmic expansions participate in the conduction chain, that it to say, that they receive and propagate the nerve impulse, contrary to Golgi’s opinion for whom these cell segments would play a merely nutritive role.
4. Excluding the substantial continuity between cell and cell, the opinion that the nervous impulse is transmitted by contact, as in the joints of electric conductors, or by a sort of induction, as in the reels of the same name, is imposed (Cajal, 1923/1981, p. 68).

Cajal also provided important data to explain neurogenesis, by studying how nerve pathways are formed. Neurons do not connect at random, but rather there is a pattern of connections that is moulded during embryonic development between the different cells. At the time there were two competing theories to try to explain how nerves grew. The *Monogenist theory*, which considered that the germ cell emitted a long prolongation from the nerve centres to the periphery, ending free, the *Polygenist theory*, which denied free growth and considered that the nerve resulted from the chain association of a multitude of cells (for review see Fernández-Santarén, 2006). Given the difficulty involved in studying an already developed adult nervous system, Cajal studied birds and mammal embryos, whose nervous system is simpler and in the process of formation, observing the evolution of the nerve cell step by step and confirming the growth of the axon end or “growth cone” (Cajal, 1890) that the *Monogenist theory* maintained thanks to the silver nitrate staining method. To explain where the growth cone moves to, Cajal postulated his *Neurotrophic Hypothesis (chemotactic hypothesis),* according to which the growth cones would be attracted to their exact location by specific substances. Cajal considered trophic factors as catalytic agents for which neurons must have specific receptors and which stimulate the growth and branching of nerve protoplasm. While this concept was not completely correct as we now know that chemorepellents appear to be as relevant as chemoattractants (De Castro et al., 2007), and the concept of neurogenesis (the process by which nervous system cells are produced by stem cells) has evolved with time and we know now that it is not restricted to prenatal stages, as neurogenesis has also been found in adults (Altman and Das, 1967), it was an absolutely revolutionary concept for the year 1892 (Cajal, 1923/1981; De Castro et al., 2007). Cajal systematically applied this rule to understand the complex structure of the brains of higher animals. Such embryological investigations were carried out by Cajal, strictly adjusting to the assumptions of Darwinian morphology and, specifically, to the law of biogenetics of Fritz Müller and Ernst Haeckel, which stated that ontogeny or individual embryonic development is a recapitulation of phylogeny or evolutionary development of the species (López-Piñero, 1986).

Also, during these years, in 1891, Cajal presented the *Principle of Dynamic Polarization* of neurons at a conference in Valencia (Cajal, 1891). Cajal objectively demonstrated the conduction capacity of dendrites and was able to explain the unidirectional transmission of the nervous impulse:

The transmission of nerve movement always takes place from the protoplasmic branches and cell body to the axon or functional expansion. Thus, every neuron posseses every neuron thus possesses a reception apparatus: the soma and the protoplasmic prolongations, an emission apparatus: the axon, and a distribution apparatus: the nerve terminal arborisation. (Cajal, 1923/1981, p. 120)

**Figure 1:**
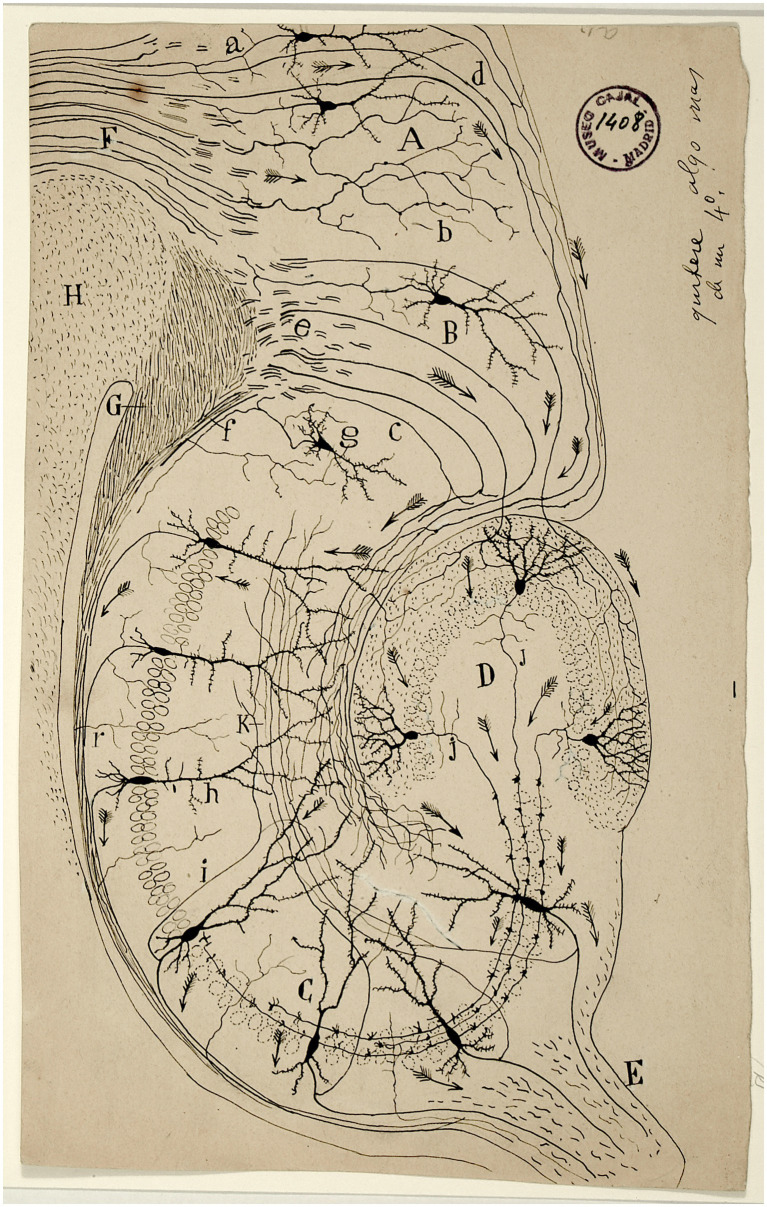
Scheme of the structure and connections of the hippocampus published by Cajal in 1901. At this time the individual neurons can be observed as well as arrows indicating the direction of the activity flow. A: occipital tip ganglion; B: subiculum; C: Ammon’s horn (CA1-CA4); D: dentate gyrus; E: fimbria; F: cingulum; G: crossed angular cord; H: corpus callosum; a: penetrating axons in the cingulum; b: cingulate fibres terminated at the occipital tip focus; c: perforating spheno-ammonium fibres; d: perforating cingulate fibres; e: plane of the superior spheno-ammonia fibres, g: subiculum cell [from Legado Cajal. Instituto Cajal (CSIC). Madrid].

In this respect, Cajal expressed in his drawings the direction of the impulse, thanks to the well-known tiny “Indian arrows,” an inference he was able to draw from the study of sensory pathways such as the olfactory or visual pathways in different species (Figure 1). However, this principle did not apply in all cases. In 1897, he realised that the soma or cell body does not always participate in the conduction of impulses. The afferent wave propagates directly from the dendrites to the axon. The *Principle of Dynamic Polarization* gave way to the *Principle of Axipetal Polarization* (Cajal, 1897).

All of these exhaustive approaches to the study of the nervous system allowed Cajal to publish his masterpiece: *Textura del sistema nervioso del hombre y de los vertebrados* (*Texture of the nervous system of man and vertebrates*), which he wrote between 1899 and 1904. This is a very relevant work not only because it refers to all of the important regions of the vertebrate nervous system, but also because it is not a simple collection of morphological descriptions, but rather attempts to tackle the physiology of the organs he studies and tries to pose the problem in terms of ontogeny and phylogeny (Fernández-Santarén, 2006). After this, a new line of research, the study of the degeneration and regeneration of the nervous system, whose results were published in 1913–1914 in the book *Estudios sobre la degeneración y regeneración del sistema nervioso (Studies on the degeneration and regeneration of the nervous system,* Cajal, 1913–1914*)* allowed Cajal to corroborate the neuronal theory once again. With his studies Cajal demonstrated that the neo-formed fibres that appear in the peripheral end of a severed nerve originate from the proliferation of the axons of the central end. This always happened in the peripheral nervous system, while in the central nervous system regeneration did not take place. However, as DeFelipe (2006a,b, 2007) indicated, Cajal’s own studies showed that, in the face of traumatic degeneration in the cerebral cortex of the cat, long axon cells were converted into short axon cells with collateral axon branches, and although the projection fibre was not repaired, the signal was transmitted by a new circuit to other neurons, which could explain the functional recovery after trauma (Cajal, 1913–1914). Cajal determined that the erroneous conclusions of his opponents stemmed from the use of inappropriate impregnation methods (DeFelipe, 2006a,b, 2007). He observed that axonal regeneration did not occur naturally in the central nervous system, unlike in the peripheral nervous system, and proposed that this was due to Schwann cells, which are present in the peripheral nervous system but absent in the central nervous system, and was the precursor of the therapeutic use of transplantation of these cells to promote central axonal regeneration (Fernández-Santarén, 2006).

Cajal also anticipated the importance of the short axon cell circuits which he described in centres such as the striatum, cerebellum, thalamus, etc. For him, they were related to late responses or reactions to external stimuli, such as memory and ideation processes (see Fernández-Santarén (2006). As early as 1901, Cajal related neuronal circuits as repositories of memory. He also hypothesised that learning is based on the establishment of new pathways, thanks to the branching and growth of dendritic and axonal arborisations. Thanks to mental exercise, the brain of a cultivated man would have many more interneuronal connections than those of an uneducated man (Cajal, 1901).

Despite all his efforts, the war against reticularism and its different variations seemed to be a constant in Cajal’s life. In fact, in 1901 the attacks on neuronal theory returned, this time from Albrecht Bethe, who began to publish a series of experimental articles on nerve regeneration, in which he claimed that axons regenerated from the anastomosis of multiple cells. This theory was known as the discontinuity or polygenic theory (for review see DeFelipe, 2006a,b, 2007). A year before his death, in 1933, Cajal wrote his article: *¿Neuronismo o reticularismo? Las pruebas objetivas de la unidad anatómica de las células nerviosas (Neuronism or reticularism? The objective proofs of nerve cells as anatomical units).* In it he summarised his main contributions on the subject (Cajal, 1933).

## Cajal and the idea of plasticity

Cajal introduced his concept or idea of plasticity in 1892 and used directly the term “plasticity” for first time in 1894. The concept was introduced in his first review of the organization of the nervous system in 1892 entitled *El nuevo concepto de la histología de los centros nerviosos (The new concept of the histology of nerve centres)*. Here, Cajal put forward his hypothesis of cerebral gymnastics as a mechanism for multiplying nerve connections and improving brain performance:

There is a notable increase in intellect among men dedicated to deep and continued mental exercise. Moreover, notable talent and even of a true genius can coexist with a medium or smaller sized brain than those of normal weight and dimensions. In the first case, given that new cells cannot be produced (nerve cells do not multiply as do muscle cells), it can be supposed that cerebral gymnastics will lead to the development of [dendritic processes] and [axonal] collaterals beyond that normal observed, forcing the establishment of new and more extensive intracortical connections […]. In the second case, there is nothing to prevent us from accepting that certain brains, either because they inherit prior adaptations or through other causes, offer a notable development of all kind of collaterals in compensation for the smaller number of cells […]. We must suppose that each psychic element in state of activity encompasses, in a vibratory or chemical way that still cannot be determined, a simple image of the impressions received either from the external world or from the weft of our organs (muscular sense). (Cajal, 1892, pages 361–376, 457–476, 505–520, 529–541; DeFelipe, 2006b)

In 1894 Cajal continued with the idea of cerebral gymnastics and presented it in two new works: In the abstract entitled *Consideraciones generales sobre la morfología de la célula nerviosa* at the International Medical Congress in Rome in 1894, and in The Croonian Lecture at The Royal Society in London (Cajal, 1894a,b; reviewed in De Carlos and Molnár, 2019) where he uses the words “dynamism,” “force of internal differentiation,” “adaptations (of neurons) to environmental conditions” and “plasticity” (Cajal, 1894a,b), and as DeFelipe (2007) asserts, it is likely that the term became popular after he used it. In these writings Cajal referred directly to plasticity in several paragraphs. With this in mind, Cajal speaks of his idea of plasticity and puts it in writing, and therefore, it is possible to affirm that Cajal is a pioneer in postulating this idea of a dynamic and changing nervous system. Cajal said:

The cerebral cortex has conserved its plasticity of growth, its strength of internal differentiation in order to accommodate itself to the growing and increasingly complicated needs of the struggle for life… (Cajal, 1894b, cited by DeFelipe, 2007, p. 80).

In this way, the associations already created between certain groups of cells would be notably reinforced by the multiplication of the terminal branches of the protoplasmic appendages and the nervous collaterals; but, in addition, completely new intercellular connections could be established thanks to the neoformation of collaterals and protoplasmic expansions (Cajal, 1894b, p. 466).

In fact, Cajal alluded in the Croonian Lecture directly to the possibility of plasticity been involved in intelligence (Cajal, 1894b, page 466–467; reviewed in Azmitia, 2007).

For Cajal, it was fundamental to understand how intellectual capacities could be improved through mental exercise and the acquisition of increasingly complex skills. He believed that it required not only the reinforcement of existing connections, but the emergence of new connections, through the progressive growth and branching of dendritic and axonal trees. He wrote in the *Revista de Ciencias Médicas (Journal of Medical Sciences)* in 1894:

…the cerebral cortex resembles a garden populated by innumerable trees, the pyramidal cells, which, thanks to intelligent cultivation, can multiply their branches, sink their roots further and further, and produce flowers and fruits that grow more exquisite every day. (Cajal, 1894a)

Cajal put forward his hypothesis on cerebral gymnastics, understanding it as a mechanism for multiplying connections and improving brain capacity. He developed this idea based on his observation of the increase in the complexity of the prolongations of the pyramidal cells (psychic cells or, as Cajal poetically called them, “butterflies of the soul”) throughout ontogenetic development and the phylogenetic scale. For him, cerebral gymnastics would lead to the development of new cortical expansions, which would allow new and more extensive interneuronal connections to be established (DeFelipe, 2006a,b). According to Cajal, the hypothesis of cerebral gymnastics was obviously possible because of his certainty in the neuronal theory which ensured the existence of free endings of nerve extensions (Cajal, 1892, 1894a,b). The reticular theory proposed a nervous system composed by static neurons that formed a diffuse network (Golgi, 1873, 1898), a brain that was not very malleable, in contrast to the neural theory of Cajal which maintained that neurons are dynamic and the nervous system is flexible (Cajal, 1888a). Cajal defended the plastic and vital properties of the neurons, as well as their contribution to brain functions such as memory and learning (Azmitia, 2007). For Cajal (1894a,b) brain dynamics depend on two factors. On one hand, heredity by which we receive a certain number of neurons with a certain propensity to associate. On the other hand, the influence of the environment which weakens or reinforces certain inherited points of association and establishes entirely new connections (through the progressive growth and branching of dendritic and axonal trees) improving neuronal organisation. As Kandel wrote, cited by Azmitia (2007): *this malleability of cortical architecture has profound implications…What can be formed by experience can presumably be undone by experience* (p. 403).

In addition to changes in neurons, Cajal saw that certain neuroglial cells of the cerebral cortex showed short and thick prolongations (retracted state), while other neuroglial cells showed numerous long and branched prolongations (relaxed state), and that between these two extremes there was a multitude of transient forms. This led Cajal to think that during mental work the morphology of the neuroglia varied, and he proposed the hypothesis of glial amoeboidism (Cajal, 1895). The cell ameboidism concept was previously introduced by Rabl-Rückhard in 1890 who proposed the hypothesis of amoeboidism and the mechanics of psychic processes:

It was based on the idea that the morphological variability of nerve cells could be due to a continuous amoeboid movement, and since the prolongations of nerve cells could be considered as elements of association between nerve cells, amoeboid movement would indicate changes in the connections…

As, as indicated above, the general idea at the time was that nerve circuits were rigid and non-changeable, Rabl-Rückhard’s hypothesis went virtually unnoticed. Lepine in 1894 and Duval in 1895 based on Rabl-Rückhard’s ideas develop the hypothesis of amoeboidism (see Black, 1981 for review) and Cajal entered into debate with Duval’s ideas, because while the latter thought that there would be a retraction of the neuronal prolongations during mental rest, for Cajal there would be an extension of the neuroglial prolongations that would cover the neuronal membrane, disconnecting the connections between the nerve cells (DeFelipe, 2008). This fruitful hypothesis on neuronal amoeboidism led authors such as Demoor in 1896 and Stefanowska in 1897 to study again experimentally the plasticity of neurons in the cerebral cortex. Stefanowska studied the phenomenon of neuronal plasticity proposed by Demoor in the cerebral cortex of guinea pigs and mice, but with emphasis on Cajal’s research on the importance of dendritic spines in the establishment of neuronal connections (reviewed in DeFelipe, 2008).

In his book *Textura del sistema nervioso del hombre y de los vertebrados* (Cajal, 1899–1904/2007), Cajal again put forward his histological hypothesis of mental work, based on his previous works. He was convinced that in order to understand how mental capacities could be improved by “mental exercise”, it was necessary to accept not only the possibility that there was a strengthening of pre-established connections, but also that new connections could appear, in the latter case through the progressive growth and branching of dendritic and axonal trees:

The architecture of the sensory centres of the brain, as well as that of the association pathways, is not absolutely fixed; there is a variable histological factor, to which all these infinite variations of mental work are attributable… The cerebral cortex is a theatre of numerous facts of dynamic action (Cajal, 1899–1904).

In the same book, he puts forward seven arguments in support of his hypothesis of plasticity (Cajal, 1899–1904, p. 1151):

1. During embryonic development, nerve dendrites and branches progressively extend and branch, coming into contact with an increasing number of neurons.
2. It is also a fact that the definitive adjustment of these relationships does not take place until after some trial and error, it’s being noted that before the expansions reach their destination and create stable articulations, numerous accessory branches disappear, a sort of trial associations whose existence proves the great initial mobility of the cellular arborisations.
3. In some cases, the [neuronal] expansions go astray by contracting abnormal connections
4. This growth movement of expansions continues after birth and there is a great difference in length and number of secondary and tertiary neuronal branches between the newborn child and the adult.
5. It is also plausible that such development is perfected in certain centres as a result of exercise and, on the contrary, is suspended and slowed down in uncultivated brain areas
6. Nerve section experiments prove that peripheral axons, both sensory and motor, are able to grow and arborise, restoring their connections to the skin and muscles and organising themselves in a somewhat different way.
7. Nervous pathology knows an infinite number of cases of functional restoration after serious lesions of differentiated cortical centres (restoration of speech articulation in motor aphasia […], etc.). This return to normality when nerve fibres have been disorganised can only be understood by admitting that the brain, as in severed peripheral nerves, the healthy end of the axon is capable of growing and emitting new collaterals which, running through the diseased parts, re-establish articulation with the disassociated neurons. When these have been destroyed, the neo-formed branches would go out to meet other nerve cells, to which they would imprint new functional character.

In addition to in *Textura del sistema nervioso del hombre y de los vertebrados* (1899–1904), Cajal used the term plasticity for some of his works related to degeneration and regeneration of the nervous system (at both, peripheral nervous system, and central nervous system, published in 1913 and 1914). These new Cajal’s studies showed, as indicated above, that after traumatic degeneration, long axon cells convert into short axon cells with collateral axon branches, and although the projection fibre is not repaired, the signal is transmitted by a new circuit to other neurons, explaining the functional recovery after trauma (Cajal, 1913–1914).

These 7 arguments and hypothesis had important influence and have been confirmed experimentally. For instance, Gray (1959) provided evidences demonstrating the importance of dendritic spines in establishing synaptic contacts, as well as later studies showing that spines could move, both developmentally and in response to synaptic stimulation (Fifková and Delay, 1982; Markham and Fifková, 1986). In addition, Valverde, found that light deprivation in mice for a certain period produced a reduction in the number of spines in the visual cortex, which was more pronounced in younger animals (Valverde, 1967, 1968, see also Rosenzweig et al., 1972). At present it is also clear that sports practice improves neuroplasticity (see for instance Nudo et al., 1996 or Lin et al., 2018) and many studies on degeneration and regeneration of the nervous system and their limitations has appeared (see Burke and Barnes, 2006; Péran et al., 2020).

Regarding to the exact historical origin of the use of the specific term “plasticity,” some controversy has been observed. The introduction of the term has been attributed to Jean Demoor in 1896, Ernesto Lugaro in 1906 and to Ioan Minea in 1909 (Stahnisch and Nitsch, 2002; DeFelipe, 2006a,b, 2007; Mateos-Aparicio and Rodríguez-Moreno, 2019) but, as we have seen, Cajal developed and communicated his idea of neuronal plasticity since 1892 in different writings. In fact, not only the idea of plasticity, but the very term plasticity and its concept is proposed by Cajal based on his experimental work (Cajal, 1892). A year later, in 1893, Eugenio Tanzi put forward a similar idea based on the strengthening of nerve connections, where the frequent passage of the nerve impulse through a connection produced a hypertrophy of the pathway and a lengthening of the nerve extensions (Tanzi, 1893; reviewed by DeFelipe, 2006a and Berlucchi and Buchtel, 2009). Such lengthening would cause the distance between contacts to shorten, increasing the functional capacity of the cells. For Tanzi, such dynamism is based on the reinforcement of existing connections (i.e., without increasing the number of contacts) to improve the efficiency of neural circuits, and not on the creation of new connections, as proposed by Cajal previously (Cajal, 1892; DeFelipe, 2008). Thus, while some controversy may still remain related to the author that unequivocally coined the term plasticity, the work of Cajal undoubtedly stimulated and influenced the first theories about synapses, synaptic transmission, and synaptic plasticity.

In some particular conceptual aspects, the term plasticity has been considered ambiguous by some researchers. For some authors plasticity refers exclusively to processes that have to do with memory and learning, for others it has to do with the dynamic nature of the nervous system, whether at the molecular, morphological, physiological or genetic level; and for others it has to do with the process of recovery of the nervous system after injury or trauma, but with doubts as to whether these degeneration/regeneration processes are exclusively related to plasticity or involve other mixed processes (Cajal in fact used the term plasticity and possibly stopped to use it when applied to degeneration/regeneration due to his intuition of the complexity of these processes) (For review see Stahnisch and Nitsch, 2002; DeFelipe, 2007; Berlucchi and Buchtel, 2009; Delgado-García, 2015; Ramirez and Arbuckle, 2016; Bernhardi et al., 2017; Mateos-Aparicio and Rodríguez-Moreno, 2019; and Andrade-Talavera et al., 2023). At present this concept is starting to be well established. The historical ambiguity seems to be related to what different authors have considered plasticity, as the concept, as indicated, has been defined from different points of view depending on the brain functions studied, the level of study of plasticity (whether the whole brain is considered, or synaptic connections only or even the individual cells), and additionally, different types of plasticity has been defined (Berlucchi and Buchtel, 2009). Thus, plasticity, is widely defined as the capacity of the nervous system to modify its physiology and morphology in response to experience (see for instance Bliss et al., 2014; Bernhardi et al., 2017). In this general way it is named “neural plasticity or neuroplasticity” when referring to the nervous system in general, “synaptic plasticity” when it refers to changes at the synaptic level, and “neuronal plasticity” when changes are studied at the level of individual neurons [also named “changes in intrinsic cell properties” by some authors (Bernhardi et al., 2017; Mateos-Aparicio and Rodríguez-Moreno, 2019; Andrade-Talavera et al., 2023)], been modifications of synaptic transmission between neurons (or even between neurons and glial cells), “synaptic plasticity,” regarded as the fundamental type of plasticity (Berlucchi and Buchtel, 2009; Mateos-Aparicio and Rodríguez-Moreno, 2019; Andrade-Talavera et al., 2023).

One the main historical discoveries related to synaptic plasticity has been the revelation of Long-Term Potentiation (LTP) of synaptic transmission in 1973 by Bliss and Lomo and Long-Term Depression (LTD) of synaptic transmission by Lynch et al. (1977), as the main forms of plasticity at synaptic level. In the following years, important mechanistic aspects of LTP and LTD were discovered as their frequent requirement of glutamate receptors, mainly NMDA receptors (both postsynaptic, Collingridge et al., 1983; or even presynaptic NMDAR, Rodríguez-Moreno and Paulsen, 2008; Rodríguez-Moreno et al., 2013), but also of KAR (Negrete-Díaz et al., 2007; Lyon et al., 2011) and mGluR (see for instance Martínez-Gallego et al., 2022a). In addition to the frequent involvement of glutamate receptors, synaptic plasticity has been generally described as needing an increase of cytoplasmic calcium, and protein kinases (for LTP) or protein phosphatases (for LTD, Kania et al., 2017; Mateos-Aparicio and Rodríguez-Moreno, 2020). In the last years, the involvement of astrocytes and other glial cells in plasticity has been defined (see Perea and Araque, 2007; Andrade-Talavera et al., 2016; Pérez-Rodríguez et al., 2019; Pérez-Otaño and Rodríguez-Moreno, 2019; Falcón-Moya et al., 2020; Martínez-Gallego et al., 2022b; Martínez-Gallego and Rodríguez-Moreno, 2023; see Andrade-Talavera et al., 2023; Louail et al., 2023 for reviews). Another important discovery in the field of synaptic plasticity has been the revelation of a new form of plasticity that seems to be more physiological (and that do not need to be induced by high frequency stimulation protocols) and is most probably the real learning rule for plasticity *in vivo*, the named spike timing-dependent plasticity (STDP that has been observed from insect to humans (Feldman, 2012 for review). LTP and LTD induced using STDP protocols are offering in the last years many insights related to the physiology of critical periods of plasticity during early development (neurodevelopment) in the hippocampus (Pérez-Rodríguez et al., 2019; Falcón-Moya et al., 2020) and the cortex (Martínez-Gallego et al., 2022b). Related to the link between brain plasticity and learning and memory processes, the field is at present in a very interesting moment trying first, to better demonstrate links between both phenomena and second whether plastic changes related to learning and memory are codified by groups neurons that are activated together (or neuron and glial cells) or just by changes in the properties of individual cells, i.e., whether the engram is synaptic or cellular (Bliss and Collingridge, 1993; Han et al., 2022). Finally, a direct relationship between synaptic plasticity and pain, addiction and brain diseases has been found (see for instance Woolf and Salter, 2000; Negrete-Díaz et al., 2007; Lüscher and Malenka, 2011; Kessels et al., 2013; Bliss et al., 2016, Andrade-Talavera and Rodríguez-Moreno, 2021; Louail et al., 2023).

Thus, thanks to Cajal’s and many other neuroscientists discoveries, the field of brain plasticity is firmly established, and it is a central theme of research. These days, in 2023, the field is celebrating the 50th anniversary of the discovery of LTP in 1973 (Bliss and Lomo, 1973) and it is dedicated to define more precisely the functions in which synaptic plasticity (LTP and LTD) are involved: in development, learning and memory as well as other neural functions and their role in brain diseases. No doubt the next years we will acquire new fundamental knowledge on plasticity that will produce social benefits in the spheres of Education and Healthcare and that will be used for a better everyday life.

## Author contributions

JR: Conceptualization, Investigation, Supervision, Validation, Writing – original draft. IM-G: Investigation, Validation, Writing – review & editing. AR-M: Conceptualization, Investigation, Supervision, Validation, Writing – original draft, Writing – review & editing.

## Footnotes

^1^Cajal always considered the development of scientific work as part of a fundamental process in the defense of his homeland in the European and world contexts.
